# New Coleoptera records from New Brunswick, Canada: Mordellidae and Ripiphoridae

**DOI:** 10.3897/zookeys.179.2583

**Published:** 2012-04-04

**Authors:** Reginald P. Webster, Jon D. Sweeney, Ian DeMerchant

**Affiliations:** 1 Natural Resources Canada, Canadian Forest Service - Atlantic Forestry Centre, 1350 Regent St., P.O. Box 4000, Fredericton, NB, Canada E3B 5P7

**Keywords:** Mordellidae, Ripiphoridae, new records, Canada, New Brunswick

## Abstract

Eleven species of Mordellidae are newly recorded for New Brunswick, Canada. Six of these, *Falsomordellistena discolor* (Melsheimer), *Falsomordellistena pubescens* (Fabricius), *Mordellistena ornata* (Melsheimer), *Mordellaria undulata* (Melsheimer), *Tomoxia inclusa* LeConte, and *Yakuhananomia bidentata* (Say)are new for the Maritime provinces. *Falsomordellistena pubescens* is new to Canada. *Pelecotoma flavipes* Melsheimer (family Ripiphoridae) is reported for the first time for New Brunswick and the Maritime provinces. Collection and habitat data are presented for all these species.

## Introduction

This paper treats new Coleoptera records from New Brunswick, Canada, of the families Mordellidae and Ripiphoridae. A general overview of the Mordellidae (tumbling flower beetles) was provided by Jackman and Lu (2002). Adults feed on pollen and are often found on flowers of umbelliferous (Apiaceae) and composite (Asteraceae) species. Larvae feed mainly in living herbaceous stems, decaying wood, and fungi, depending on the species (Jackman and Lu 2002). Ford and Jackman (1996) summarized the known larval host plants of North American species of this family. The North American species were revised by Liljeblad (1945), and Bright (1986) provided a catalog of North American species. Later, Jackman (1991) made additions and corrections to the catalog, and Jackman and Lu (2001) and Lisberg (2003) made additional taxonomic changes to North American species. McNamara (1991) reported 14 mordellid species and subspecies for New Brunswick. Majka and Jackman (2006), in a review of the Mordellidae of the Maritime provinces (New Brunswick, Nova Scotia, Prince Edward Island), reported another six species for New Brunswick and removed *Mordella atrata lecontei* Csiki, bringing the total number of known species to 19. Here, we add 11 species to the faunal list of the province.

Falin (2002) provided a general review of the Ripiphoridae (the ripiphorid beetles) of North America, including an overview of the biology and life history of various members of this family. Members of this family are unusual among Coleoptera in that they are endoparasitoids on insects such as aculeate Hymenoptera, Coleoptera (Anobiidae, Cerambycidae), and Orthoptera (Blattidae) (Falin 2002). Species occurring in the Maritime provinces are parasitoids of insects such as aculeate Hymenoptera (Apidae, Halictidae) (*Ripiphorus* sp.) and beetles in the genus *Ptilinus* (*Pelecotoma*) (Linsley et al. 1952; Svácha 1994; Falin 2002). Campbell (1991) reported 10 species of Ripiphoridae from Canada but none from the Maritime provinces. Majka et al. (2006) reported *Ripiphorus fasciatus* (Say) for the first time for New Brunswick and Nova Scotia and the Maritime provinces. William McIntosh (former director of the New Brunswick Museum) in an unpublished manuscript reported a specimen of *Ripiphorus zeschii* (LeConte) (determined by W. H. Harrington) from Saint John, collected sometime between 1898 and 1907 (Majka et al. 2006). To date, no specimen has been found to support this record and this record is considered questionable. Here, we report another species of Ripiphoridae for New Brunswick and the Maritime provinces.

## Methods and conventions

The following records are based on specimens collected during a general survey by the first author to document the Coleoptera fauna of New Brunswick and from by-catch samples obtained during a study to develop a general attractant for the detection of invasive species of Cerambycidae. Additional records were obtained from specimens contained in the collection belonging to Natural Resources Canada, Canadian Forest Service - Atlantic Forestry Centre, Fredericton, New Brunswick.

### Collection methods

Many specimens of Mordellidae were collected by sweeping vegetation or flowers. Others were collected from Lindgren 12-funnel trap samples during a study to develop a general attractant for the detection of invasive species of Cerambycidae. These traps visually mimic tree trunks and are often effective for sampling species of Coleoptera that live in microhabitats associated with standing trees (Lindgren 1983). See Webster et al. (in press) for details of the methods used for trap deployment and sample collection. A description of the habitat was recorded for all specimens collected during this survey. Locality and habitat data are presented exactly as on labels for each record. This information, as well as additional collecting notes, is summarized and discussed in the collection and habitat data section for each species.

### Distribution

Distribution maps, created using ArcMap and ArcGIS, are presented for each species in New Brunswick. Every species is cited with current distribution in Canada and Alaska, using abbreviations for the state, provinces, and territories. New records for New Brunswick are indicated in bold under Distribution in Canada and Alaska. The following abbreviations are used in the text:

**Table:** 

**AK**	Alaska	**MB**	Manitoba
**YT**	Yukon Territory	**ON**	Ontario
**NT**	Northwest Territories	**QC**	Quebec
**NU**	Nunavut	**NB**	New Brunswick
**BC**	British Columbia	**PE**	Prince Edward Island
**AB**	Alberta	**NS**	Nova Scotia
**SK**	Saskatchewan	**NF & LB**	Newfoundland and Labrador

Acronyms of collections examined or where specimens reside referred to in this study are as follows:

**AFC** Atlantic Forestry Centre, Natural Resources Canada, Canadian Forest Service, Fredericton, New Brunswick, Canada

**CGMC** Christopher G. Majka Collection, Halifax, Nova Scotia, Canada

**CNC** Canadian National Collection of Insects, Arachnids and Nematodes, Agriculture and Agri-Food Canada, Ottawa, Ontario

**NBM** New Brunswick Museum, Saint John, New Brunswick, Canada

**RWC** Reginald P. Webster Collection, Charters Settlement, New Brunswick, Canada

## Results

### Species accounts

All records below are species newly recorded for New Brunswick, Canada. Species followed by ** are newly recorded from the Maritime provinces of Canada.

The classification of the Mordellidae and Ripiphoridae follows Bouchard et al. (2011).

### Family Mordellidae Latreille, 1802

Majka and Jackman (2006) newly reported six species of Mordellidae for the province of New Brunswick and removed *Mordella atrata lecontei* Csiki, bringing the total number of known species to 19. Eleven more species are reported here (Table 1). *Mordellaria undulata* (Melsheimer), *Tomoxia inclusa* LeConte, *Yakuhananomia bidentata* (Say), *Falsomordellistena discolor* (Melsheimer), *Falsomordellistena pubescens* (Fabricius), and *Mordellistena ornata* (Melsheimer)are new for the Maritime provinces.

**Table 1.** Species of Tenebrionidae and Zopheridae recorded from New Brunswick, Canada.

**Family Mordellidae Latreille**
**Subfamily Mordellinae Latreille**
**Tribe Mordellini Latreille**
*Mordella atrata atrata* Melsheimer
*Mordella marginata marginata* Melsheimer
*Mordella melaena* Germar
*Mordellaria borealis* (LeConte)
*Mordellaria serval* (Say)
*Mordellaria undulata* (Melsheimer)**
*Tomoxia inclusa* LeConte**
*Tomoxia lineella* LeConte
*Yakuhananomia bidentata* (Say)**
**Tribe Mordellistenini Ermisch**
*Falsomordellistena discolor* (Melsheimer)**
*Falsomordellistena pubescens* (Fabricius)***
*Glipostenoda ambusta* (LeConte)*
*Mordellina ancilla* (LeConte)*
*Mordellina infima* (LeConte)
*Mordellina nigricans* (Melsheimer)
*Mordellina pustulata* (Mlesheimer)*
*Mordellistena aspersa* (Melsheimer)
*Mordellistena cervicalis* LeConte
*Mordellistena errans* Fall
*Mordellistena frosti* Liljeblad
*Mordellistena fuscipennis* (Melsheimer)*
*Mordellistena indistincta* Smith
*Mordellistena limbalis* (Melsheimer)
*Mordellistena marginalis* (Say)
*Mordellistena ornata* (Melsheimer)**
*Mordellistena picilabris* Helmuth*
*Mordellistena syntaenia* Liljeblad
*Mordellistena tosta* LeConte
*Mordellistena trifasciata* (Say)
*Mordellochroa scapularis* (Say)
**Family Rhipiphoridae Gemminger**
**Subfamily Pelecotominae Seidlitz**
*Pelecotoma flavipes* Melsheimer**
**Subfamily Ripiphorinae Gemminger**
**Tribe Ripiphorini Gemminger**
*Ripiphorus fasciatus* (Say)

**Notes:** *New to province; **New to Maritime provinces.

### Subfamily Mordellinae Latreille, 1802

**Tribe Mordellini Latreille, 1802**

#### Mordellaria undulata

(Melsheimer, 1846)**

http://species-id.net/wiki/Mordellaria_undulata

Map 1

##### Material examined.

**New Brunswick, Queens Co.**, Cranberry Lake P.N.A. (Protected Natural Area), 46.1125°N, 65.6075°W, 4–18.VIII.2011, M. Roy & V. Webster, Lindgren funnel traps in forest canopy (2, RWC).

**Map 1.**
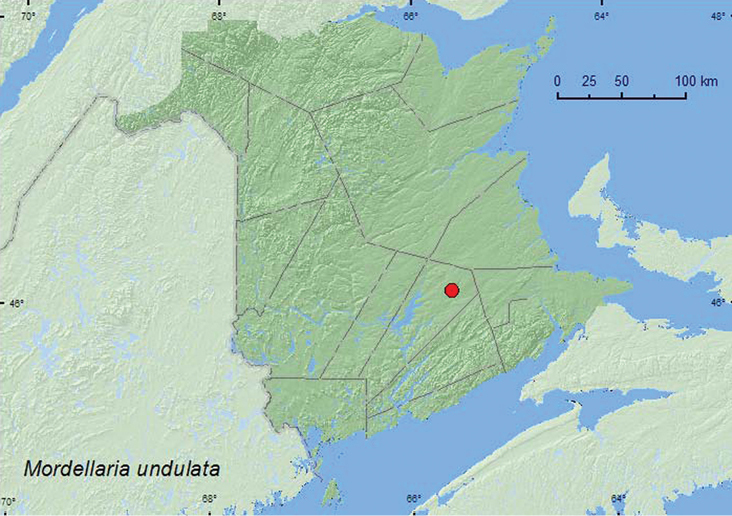
Collection localities in New Brunswick, Canada of *Mordellaria undulata*.

##### Collection and habitat data.

The adults in New Brunswick were captured in Lindgren funnel traps deployed in the forest canopy in a red oak (*Quercus rubra* L.) forest. Adults were collected during July and August. This species has been beaten from dead limbs of various species of hardwoods in Indiana (Downie and Arnett 1996).

##### Distribution in Canada and Alaska.

ON, **NB** (McNamara 1991).

#### Tomoxia inclusa

LeConte, 1862**

http://species-id.net/wiki/Tomoxia_inclusa

Map 2

##### Material examined.

**New Brunswick, Queens Co.**, Cranberry Lake P.N.A, 46.1125°N, 65.6075°W, 6.VIII.2009, M.-A. Giguère, old red oak forest, on flowers of *Spiraea alba* (1, AFC); same locality data and forest type, 13–20.VII.2011, M. Roy & V. Webster, Lindgren funnel trap in forest canopy (1, RWC). **Sunbury Co.**, Acadia Research Forest, 45.9866°N, 66.3841°W, 21–29.VII.2009, R. Webster & M.-A. Giguère, mature (110-year-old) red spruce forest with scattered red maple and balsam fir, Lindgren funnel trap (1, AFC). **York Co.**, Charters Settlement, 45.8267°N, 66.7343°W, 8.VII.2005, R. P. Webster, mixed forest, on recently cut spruce log (1, RWC); same locality and collector but 45.8331°N, 66.7410°W, 23.VII.2005, sedge marsh on flowers of *Spiraea alba* (1, RWC).

**Map 2.**
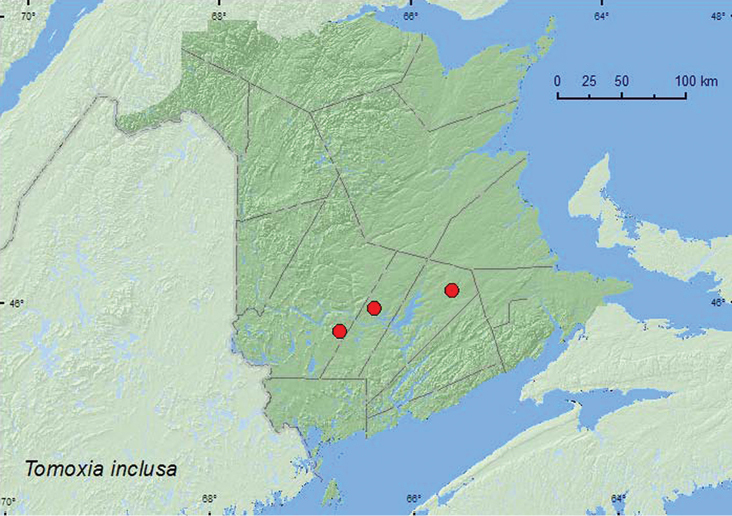
Collection localities in New Brunswick, Canada of *Tomoxia inclusa*.

##### Collection and habitat data.

*Tomoxia inclusa* was found in an old red oak forest, a mixed forest, and a mature red spruce (*Picea rubens* Sarg.) forest. Adults were collected from flowers of meadowsweet (*Spiraea alba* Du Roi), on a recently cut spruce (*Picea* sp.) log, and in Lindgren funnel traps. This species was collected during July in New Brunswick. This species has been reared from *Tilia* sp. (Brimley 1951).

##### Distribution in Canada and Alaska.

ON, QC, **NB** (McNamara 1991).

#### Yakuhananomia bidentata

(Say, 1824)**

http://species-id.net/wiki/Yakuhananomia_bidentata

Map 3

##### Material examined.

**New Brunswick, Queens Co.**, Grand Lake Meadows P.N.A., 45.8227°N, 66.1209°W, 5–19.VII.2011, 19.VII-5.VIII.2011, M. Roy & V. Webster, old silver maple forest and seasonally flooded marsh, Lindgren funnel traps in forest canopy (4, RWC).

**Map 3.**
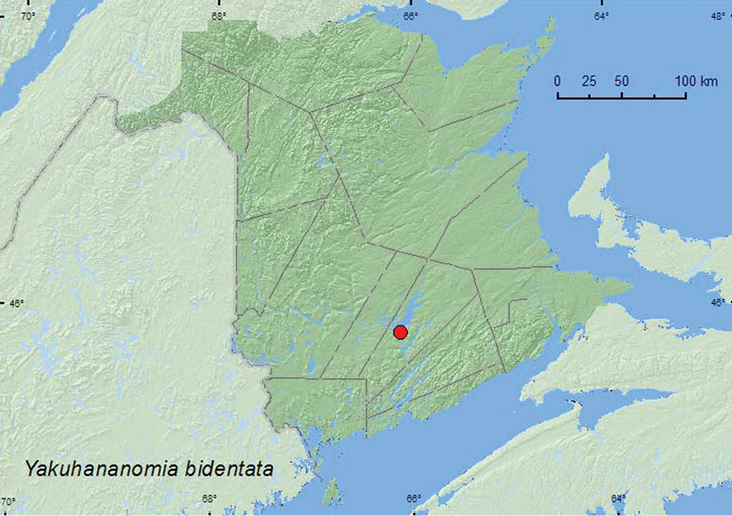
Collection localities in New Brunswick, Canada of *Yakuhananomia bidentata*.

##### Collection and habitat data.

In New Brunswick, adults of this large species were captured during July and August in Lindgren funnel traps in the forest canopy in a silver maple (*Acer saccharinum* L.) swamp. Downie and Arnett (1996) reported this species from boles of dead and dying hickory (*Carya* sp.) trees in Indiana and New York.

##### Distribution in Canada and Alaska.

ON, QC, **NB** (McNamara 1991).

### Tribe Mordellistenini Ermisch, 1941

#### Falsomordellistena discolor

(Melsheimer, 1846)**

http://species-id.net/wiki/Falsomordellistena_discolor

Map 4

##### Material examined.

**New Brunswick, Queens Co.**, Cranberry Lake P.N.A, 46.1125°N, 65.6075°W, 28.VII-6.VIII.2009, R. Webster & M.-A. Giguère, old red oak forest, Lindgren funnel traps (2, RWC); same locality data and forest type, 29.VI–7.VII.2011, 7–14.VII.2011, 20.VII–4.VIII.2011, M. Roy & V. Webster, Lindgren funnel traps in forest canopy (29, AFC, NBM, RWC). **York Co.**, Charters Settlement, 45.8300°N, 66.7347°W, 29.VIII.2004, R. P. Webster, regenerating mixed forest, on foliage (1, RWC).

**Map 4.**
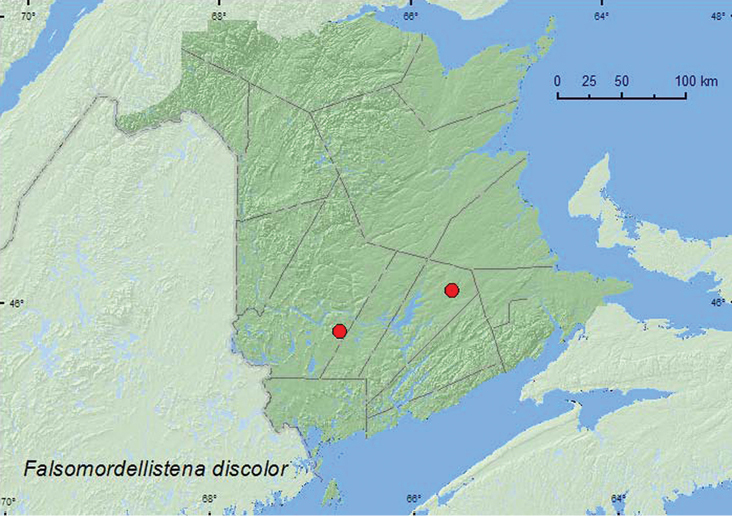
Collection localities in New Brunswick, Canada of *Falsomordellistena discolor*.

##### Collection and habitat data.

A large series of this species was captured in Lindgren funnel traps deployed in the forest canopy of an old red oak forest. One individual was found on foliage in a regenerating (20-year-old) mixed forest. Adults were captured during July and August. In Wisconsin, this species was captured in flight intercept traps and malaise traps in sandy oak barrens and a mixed southern forest (Lisberg and Young 2003).

##### Distribution in Canada and Alaska. 

ON, **NB** (McNamara 1991).

#### Falsomordellistena pubescens

(Fabricius, 1798)***

http://species-id.net/wiki/Falsomordellistena_pubescens

Map 5

##### Material examined.

**Canada, New Brunswick,** **York Co.**, Rt. 645 at Beaver Brook, 45.6830°N, 66.8679°W, 8.VII.2008, R. P. Webster, red maple and alder swamp, on flowers of *Ilex verticiliata* (winter berry) (18, NBM, RWC).

**Map 5.**
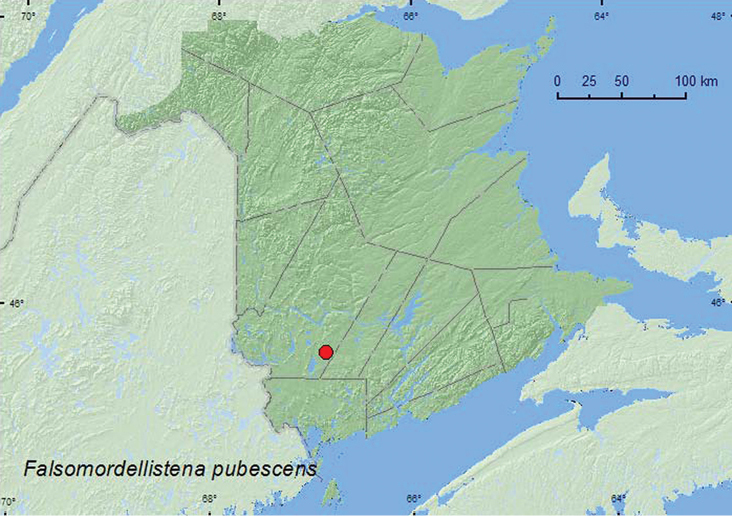
Collection localities in New Brunswick, Canada of *Falsomordellistena pubescens*.

##### Collection and habitat data.

A large series of this species was collected from flowers of winter berry (*Ilex verticiliata* (L.) Gray) in a red maple (*Acer rubrum* L.) and alder (*Alnus* sp.) swamp. Adults were captured during early July. In Wisconsin, this species was captured in malaise traps on the margin of a southern mixed deciduous hardwood forest (Lisberg and Young 2003). Downie and Arnett (1996) reported this species as common on various wild flowers in Indiana.

##### Distribution in Canada and Alaska.

**NB** (new Canadian record).

#### Glipostenoda ambusta

(LeConte, 1862)

http://species-id.net/wiki/Glipostenoda_ambusta

Map 6

##### Material examined.

**New Brunswick, Carleton Co.**,Jackson Falls,Bell Forest, 46.2200°N, 67.7231°W, 19–28.VII.2008, R. P. Webster, mature hardwood forest, Lindgren funnel trap (1, RWC).

**Map 6.**
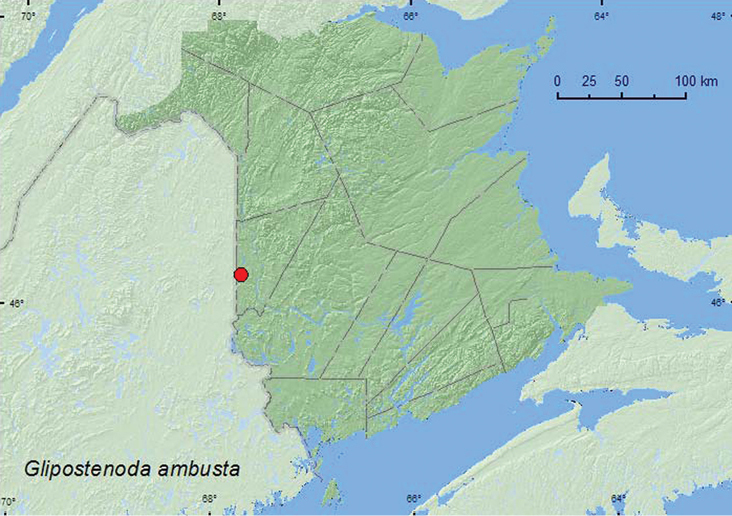
Collection localities in New Brunswick, Canada of *Glipostenoda ambusta*.

##### Collection and habitat data.

One individual of this species was captured during July in a Lindgren funnel trap in a mature hardwood forest with sugar maple (*Acer saccharum* Marsh.), American beech (*Fagus grandifolia* Ehrh.), and white ash (*Fraxinus americana* L.). Elsewhere, Lisberg and Young (2003) collected one specimen of this species from Queen Anne’s lace (*Daucus carota* L.) and others from flight intercept and malaise traps in an oak barrens adjacent to a sand barrens and field. Downie and Arnett (1996) reported this species from basswood (*Tilia americana* L.) in Indiana.

##### Distribution in Canada and Alaska.

BC, ON, QC, **NB,** NS (McNamara 1991; Majka and Jackman 2006). Majka and Jackman (2006) reported this species for the first time in the Maritime provinces from Nova Scotia.

#### Mordellina ancilla

(LeConte, 1862)

http://species-id.net/wiki/Mordellina_ancilla

Map 7

##### Material examined.

**New Brunswick, York Co.**, Charters Settlement, 45.8430°N, 66.7275°W, 27.VI.2004, R. P. Webster, regenerating mixed forest, sweeping foliage in brushy opening (1, RWC).

**Map 7.**
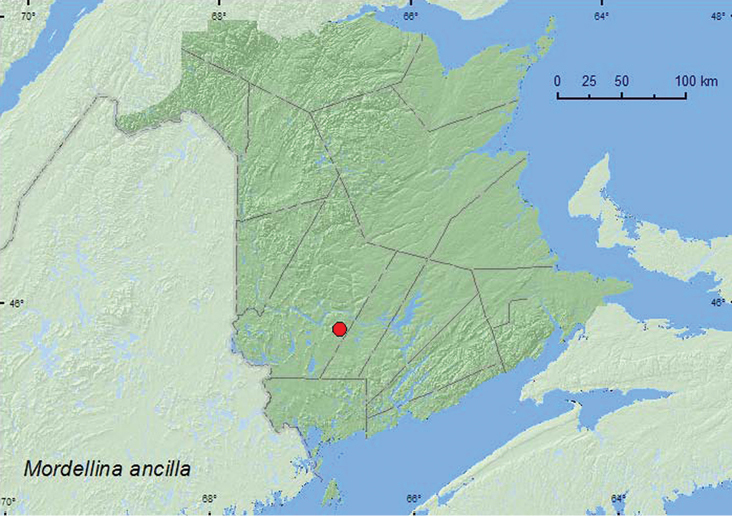
Collection localities in New Brunswick, Canada of *Mordellina ancilla*.

##### Collection and habitat data.

One individual of this species was swept from foliage in a brushy opening of a regenerating (20-year-old) mixed forest during late June. This species was reared from *Gleditsia triacanthos* L. (Fabaceae) (larvae feed inside the thorns) in Tennessee, USA (Ford and Jackman 1996), but undoubtedly uses other hosts in New Brunswick as this host species does not occur in the province.

##### Distribution in Canada and Alaska.

ON, **NB,** NS (McNamara 1991; Majka and Jackman 2006). *Mordellina ancilla* was newly recorded from Nova Scotia and the Maritime provinces by Majka and Jackman (2006).

#### Mordellina pustulata

(Melsheimer, 1846)

http://species-id.net/wiki/Mordellina_pustulata

Map 8

##### Material examined.

**New Brunswick, Carleton Co.**, Meduxnekeag Valley Nature Preserve, 46.1931°N, 67.6825°W, 13.VII.2004, K. Bredin, J. Edsall, & R. Webster, river margin, sweeping foliage (1, RWC). **York Co.**, Charters Settlement, 45.8430°N, 66.7275°W, 20.VII.2008, R. P. Webster, old field within regenerating mixed forest, sweeping flowers of *Aralia hispida* (5, RWC); same locality data and collector but 30.VII.2008, regenerating mixed forest, sweeping foliage in brushy opening (1, RWC). **Sunbury Co.**, ca. 2.5 km S of Beaver Dam, 45.7703°N, 66.6867°W, 26.VI.2007, R. P. Webster, mixed forest with red pine, along power-line cut, sweeping foliage (1, RWC).

**Map 8.**
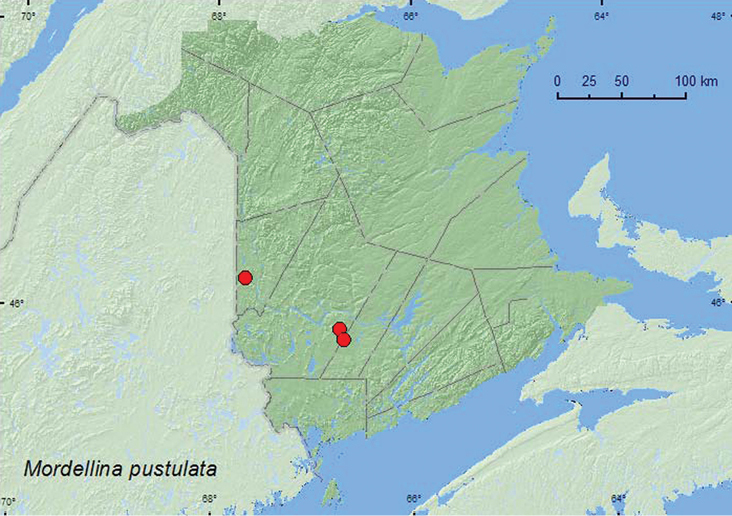
Collection localities in New Brunswick, Canada of *Mordellina pustulata*.

##### Collection and habitat data.

In New Brunswick, *Mordellina pustulata* adults were swept from foliage along a river margin, in a regenerating (20-year-old) mixed forest, and along a power-line right-of-way through a mixed forest. Adults were also swept from flowers of bristly sarsaparilla (*Aralia hispida* Vent.) in a small old field within a regenerating (20-year-old) mixed forest. This species was collected during late June and July. Elsewhere, *Mordellina pustulata* has been reared from stems of *Gentiana andrewsii* Griseb. (Gentianaceae), *Veronia altissima* Nutt. (Scrophulariaceae), and 19 species of Asteraceae (Ford and Jackman 1996; Lisberg and Young 2003).

##### Distribution in Canada and Alaska.

BC, AB, SK, MB, ON, QC, **NB**, NS (McNamara 1991; Majka and Jackman 2006).This species was newly recorded from Nova Scotia and the Maritime provinces by Majka and Jackman (2006).

#### Mordellistena fuscipennis

(Melsheimer, 1846)

http://species-id.net/wiki/Mordellistena_fuscipennis

Map 9

##### Material examined. 

**New Brunswick, Carleton Co.**,Jackson Falls,Bell Forest, 46.2200°N, 67.7231°W, 19–28.VII.2008, 28.VII-6.VIII.2008, 6–14.VIII.2008, R. P. Webster, mature hardwood forest, Lindgren funnel traps (5, AFC, RWC); same locality and forest type, 31.VII-7.VIII.2009, 7–12.VIII.2009, R. Webster & M.-A. Giguère, Lindgren funnel trap (2, RWC). **Queens Co.**, Cranberry Lake P.N.A, 46.1125°N, 65.6075°W, 28.VII-6.VIII.2009, 6–14.VIII.2009, R. Webster & M.-A. Giguère, old red oak forest, Lindgren funnel traps (2, AFC, RWC). **Restigouche Co.**, Dionne Brook P.N.A., 47.9030°N, 68.3503°W, 9–23.VIII.2011, M. Roy & V. Webster, old-growth northern hardwood forest, Lindgren funnel trap (1, NBM); same locality and collectors but 47.9064°N, 68.3441°W, 28.VII-9.VIII.2011, old-growth white spruce and balsam fir forest, Lindgren funnel trap (1, NBM). **Sunbury Co.**, Acadia Research Forest, 45.9866°N, 66.3841°W, 21–29.VII.2009, R. Webster & M.-A. Giguère, mature (110-year-old) red spruce forest with scattered red maple and balsam fir, Lindgren funnel trap (1, AFC). **York Co.**, Charters Settlement, 45.8430°N, 66.7275°W, 28.VIII.2004, R. P. Webster, mixed forest, on goldenrod (1, RWC); same locality and collector, 45.8395°N, 66.7391°W, 23.VII.2007, 5.VIII.2009, mixed forest, u.v. light and Lindgren funnel traps (3, NBM, RWC); 15 km W of Tracy off Rt. 645, 45.6848°N, 66.8821°W, 20–29.VII.2009, R. Webster & M.-A. Giguère, old red pine forest, Lindgren funnel trap (1, AFC); 14 km WSW of Tracy, S of Rt. 645, 45.6741°N, 66.8661°W, 13–27.VII.2010, R. Webster & C. MacKay, old mixed forest with red and white spruce, red and white pine, balsam fir, eastern white cedar, red maple, and *Populus* sp., Lindgren funnel trap (1, AFC).

**Map 9.**
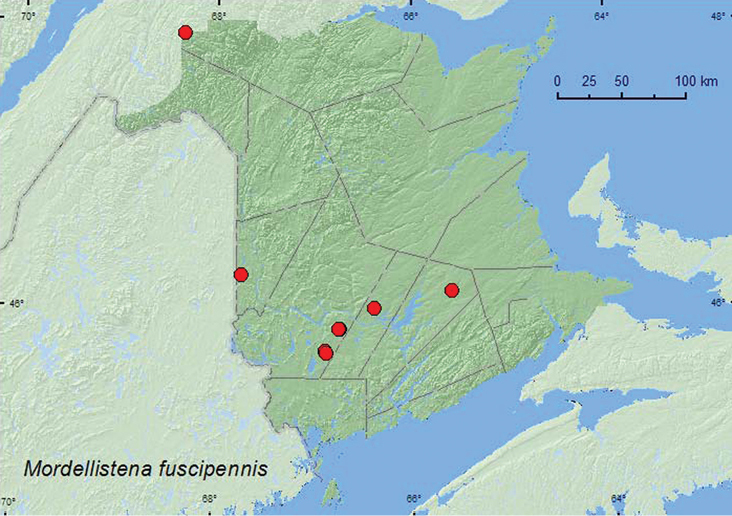
Collection localities in New Brunswick, Canada of *Mordellistena fuscipennis*.

##### Collection and habitat data.

In Wisconsin, adults of *Mordellistena fuscipennis* were collected at a black light and from flight intercept and malaise traps in various hardwood forests (Lisberg and Young 2003). In Nova Scotia, adults were collected from deciduous and mixed coniferous forests (Majka and Jackman (2006). This species was also found in various deciduous and conifer forest types in New Brunswick. Adults were captured in Lindgren funnel traps in a mature hardwood forest with sugar maple, American beech, and white ash, an old red oak forest, an old-growth northern hardwood forest with sugar maple and yellow birch (*Betula alleghaniensis* Britt.), in old and mature mixed forests, in an old red pine (*Pinus resinosa* Ait.) forest, in a mature red spruce forest, and in an old-growth white spruce (*Picea glauca* (Moench) and balsam fir (*Abies balsamea* (L.) Mill.) forest. The only adult with specific micro-habitat data was collected from goldenrod (*Solidago* sp.) flowers. Two individuals were collected at an ultraviolet light near a mixed forest. Adults were collected during July and August.

##### Distribution in Canada and Alaska.

ON, QC, **NB**, PE, NS, (McNamara 1991; Majka and Jackman 2006).

#### Mordellistena ornata

(Melsheimer, 1846)**

http://species-id.net/wiki/Mordellistena_ornata

Map 10

##### Material examined.

**New Brunswick,** **Carleton Co.**,Jackson Falls,Bell Forest, 46.2152°N, 67.7190°W, 12.VII.2004, K. Bredin, J. Edsall, & R. Webster, river margin, sweeping foliage (1, RWC). **Queens Co.**, Grand Lake Meadows P.N.A., 45.8227°N, 66.1209°W, 15–29.VI.2009, R. Webster & C. MacKay, old silver maple forest with green ash and seasonally flooded marsh, Lindgren funnel trap (1, AFC).

**Map 10.**
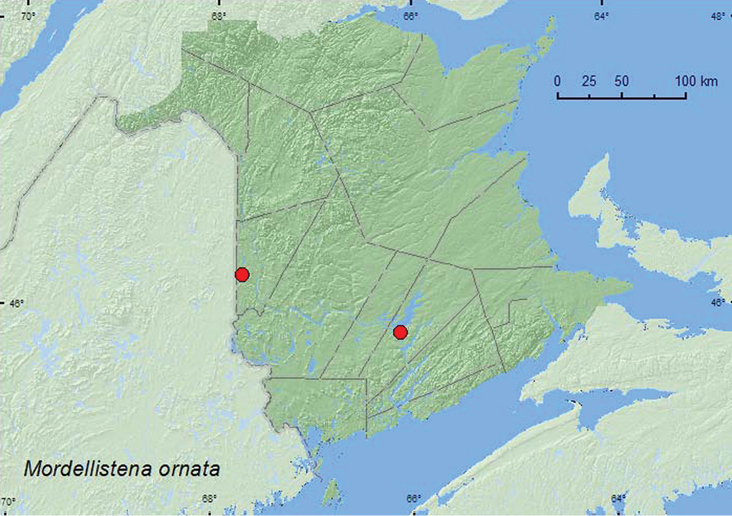
Collection localities in New Brunswick, Canada of *Mordellistena ornata*.

##### Collection and habitat data.

In Wisconsin, *Mordellistena ornata* was collected from sumac (*Rhus* sp.), poplar (*Populus* sp.), *Ceanothus* sp., and flowers of two *Cornus* species (Lisberg and Young 2003). Adults were also captured in flight intercept and malaise traps in oak forests, and in southern and northern hardwood forests. In New Brunswick, this species was swept from foliage along a river margin and captured in a Lindgren funnel trap deployed in an old silver maple swamp. Adults were captured during June and July.

##### Distribution in Canada and Alaska.

SK, MB, ON, QC, **NB** (McNamara 1991).

#### Mordellistena picilabris

Helmuth, 1864

http://species-id.net/wiki/Mordellistena_picilabris

Map 11

##### Material examined.

**New Brunswick, Albert Co.**, Mary’s Point, 21.V.2005, C. G. Majka, salt marsh, on flowers (1, CGMC). **Restigouche Co.**, Blackland, 22.VIII.2007, J. S. McIvor, in grass (1, CGMC). **Sunbury Co.**, 9.5 km NE of jct. 101 & 645, 45.7586°N, 66.6755°W, 17.VII.2008, R. P. Webster, old field with open sandy areas, sweeping foliage (1, RWC). **York Co.**, Charters Settlement, 45.8340°N, 66.7450°W, 10.VII.2005, R. P. Webster, old field, sweeping (1, RWC); same locality and collector but 45.8430°N, 66.7275°W, 20.VII.2008, old field within regenerating (20 years-old) mixed forest, seeping flowers of *Aralia hispida* (1, RWC).

**Map 11.**
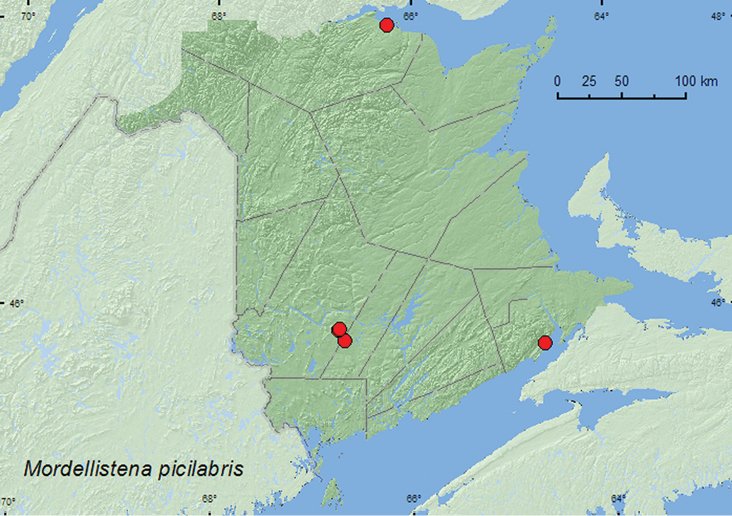
Collection localities in New Brunswick, Canada of *Mordellistena picilabris*.

##### Collection and habitat data.

In New Brunswick, *Mordellistena picilabris* was collected from flowers in a salt marsh, from grass, and by sweeping foliage and *Aralia hispida* in old fields. Adults were captured during May, July, and August.

##### Distribution in Canada and Alaska.

MB, **NB**, NS, PE (McNamara 1991; Majka and Jackman 2006). Majka and Jackman (2006) reported this species as newly recorded for New Brunswick, Nova Scotia, and Prince Edward Island in Table 1 of their list of species of the Maritime provinces but inadvertently included no supporting data for the New Brunswick record. Here, we include this record and additional records that establish the presence of this species for New Brunswick.

### Family Rhipiphoridae Gemminger, 1870

Majka et al. (2006) reported *Ripiphorus fasciatus* (Say) and the family Rhipiphoridae for the first time for New Brunswick, Nova Scotia, and the Maritime provinces. Here, we report another species of Ripiphoridae, *Pelecotoma flavipes* Melsheimer, for the first time for New Brunswick and the Maritime provinces (Table 1).

### Subfamily Pelecotominae Seidlitz, 1875

#### Pelecotoma flavipes

Melsheimer, 1846**

http://species-id.net/wiki/Pelecotoma_flavipes

Map 12

##### Material examined.

**New Brunswick, Queens Co.**, Cranberry Lake P.N.A, 46.1125°N, 65.6075°W, 10–15.VII.2009, R. Webster & M.-A. Giguère, old red oak forest, Lindgren funnel traps (2, RWC); same locality data and forest type, 7–13.VII.2011, 13–20.VII.2011, M. Roy & V. Webster, Lindgren funnel traps in forest canopy (13, AFC, NBM, RWC). **Victoria Co.**, Arthurette, (no day given).III.1959, C. C. Smith, emerged from barn timbers (1, AFC). **York Co.**, 15 km W of Tracy off Rt. 645, 45.6848°N, 66.8821°W, 30.VI–13.VII.2010, R. Webster & K. Burgess, old red pine forest, Lindgren funnel trap (in forest canopy) (1, AFC).

**Map 12.**
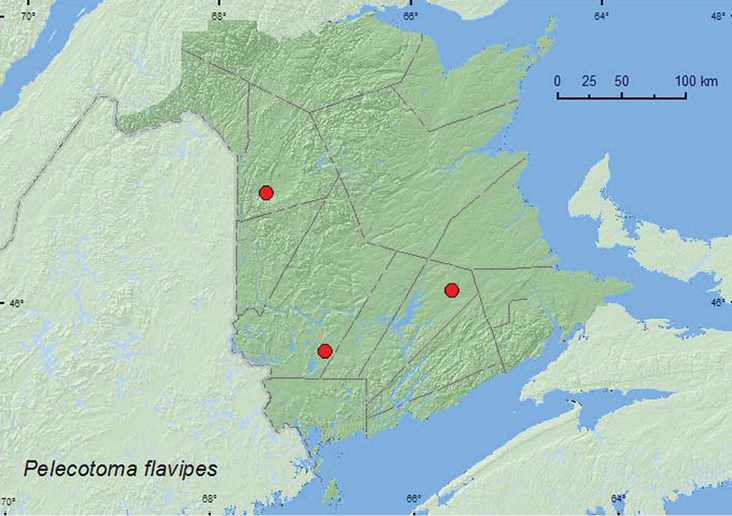
Collection localities in New Brunswick, Canada of *Pelecotoma flavipes*.

##### Collection and habitat data.

A large series of this species was captured in Lindgren funnel traps in an old red oak forest. Most individuals were captured in traps in the forest canopy. One individual was captured in a Lindgren funnel trap in the canopy of a red pine in an old red pine forest; another emerged from barn timbers. Adults were captured during July. This species is a parasitoid of *Ptilinus ruficornis* (Say) (Anobiidae) and is found on exposed dead wood of sugar maples, American beech, and oak (Stephens 1968; Acciavatti and Simone 1976) and probably has a life history similar to that of *Pelecotoma fennica* (Svácha 1994). *Ptilinus ruficornis* was common at both localities where *Pelecotoma flavipes* was collected.

##### Distribution in Canada and Alaska.

ON, QC, **NB** (Campbell 1991)

## Supplementary Material

XML Treatment for Mordellaria undulata

XML Treatment for Tomoxia inclusa

XML Treatment for Yakuhananomia bidentata

XML Treatment for Falsomordellistena discolor

XML Treatment for Falsomordellistena pubescens

XML Treatment for Glipostenoda ambusta

XML Treatment for Mordellina ancilla

XML Treatment for Mordellina pustulata

XML Treatment for Mordellistena fuscipennis

XML Treatment for Mordellistena ornata

XML Treatment for Mordellistena picilabris

XML Treatment for Pelecotoma flavipes
